# Overexpression of stathmin in breast carcinomas points out to highly proliferative tumours

**DOI:** 10.1054/bjoc.1999.0891

**Published:** 1999-12-08

**Authors:** P A Curmi, C Noguès, S Lachkar, N Carelle, M-P Gonthier, A Sobel, R Lidereau, I Bièche

**Affiliations:** INSERM U440, IFM, 17 rue du Fer à Moulin, Paris, 75005, France; Laboratoire d'Oncogénétique, Centre René Huguenin, 35 rue Dailly, St-Cloud, 92211, France

**Keywords:** human breast cancer, stathmin, quantitative RT-PCR, protein phosphorylation, prognostic factors, DNA

## Abstract

We recently discovered that stathmin was overexpressed in a subgroup of human breast carcinomas. Stathmin is a cytosolic phosphoprotein proposed to act as a relay integrating diverse cell signalling pathways, notably during the control of cell growth and differentiation. It may also be considered as one of the key regulators of cell division for its ability to destabilize microtubules in a phosphorylation-dependent manner. To assess the significance of stathmin overexpression in breast cancer, we evaluated the correlation of stathmin expression, quantified by reverse transcription polymerase chain reaction, with several disease parameters in a large series of human primary breast cancer (*n* = 133), obtained in strictly followed up women, whose clinico-pathological data were fully available. In agreement with our preliminary survey, stathmin was found overexpressed in a subgroup of tumours (22%). In addition, overexpression was correlated to the loss of steroid receptors (oestrogen, *P* = 0.0006; progesterone, *P* = 0.008), and to the Scarff–Bloom–Richardson histopathological grade III (*P* = 0.002), this latter being ascribable to the mitotic index component (*P* = 0.02). Furthermore studies at the DNA level indicated that stathmin is overexpressed irrespective of its genomic status. Our findings raise important questions concerning the causes and consequences of stathmin overexpression, and the reasons of its inability to counteract cell proliferation in the overexpression group. © 2000 Cancer Research Campaign


*British Journal of Cancer* (2000) **82**(1), 142–150
© 2000 Cancer Research Campaign
Article no. bjoc.1999.0891

### Overexpression of stathmin in breast carcinomas points out to highly proliferative tumours

**PA Curmi[1], C Noguès[2], S Lachkar[1], N Carelle[2], M-P Gonthier[1], A Sobel[1], R Lidereau[2] and I Bièche[2]**

[1]INSERM U440, IFM, 17 rue du Fer à Moulin, 75005 Paris, France; [2] Laboratoire d'Oncogénétique, Centre René Huguenin, 35 rue Dailly, 92211 St-Cloud, France

**Summary** We recently discovered that stathmin was overexpressed in a subgroup of human breast carcinomas. Stathmin is a cytosolic phosphoprotein proposed to act as a relay integrating diverse cell signalling pathways, notably during the control of cell growth and differentiation. It may also be considered as one of the key regulators of cell division for its ability to destabilize microtubules in a phosphorylation-dependent manner. To assess the significance of stathmin overexpression in breast cancer, we evaluated the correlation of stathmin expression, quantified by reverse transcription polymerase chain reaction, with several disease parameters in a large series of human primary breast cancer ($n$ = 133), obtained in strictly followed up women, whose clinico-pathological data were fully available. In agreement with our preliminary survey, stathmin was found overexpressed in a subgroup of tumours (22%). In addition, overexpression was correlated to the loss of steroid receptors (oestrogen, $P$ = 0.0006; progesterone, $P$ = 0.008), and to the Scarff–Bloom–Richardson histopathological grade III ($P$ = 0.002), this latter being ascribable to the mitotic index component ($P$ = 0.02). Furthermore studies at the DNA level indicated that stathmin is overexpressed irrespective of its genomic status. Our findings raise important questions concerning the causes and consequences of stathmin overexpression, and the reasons of its inability to counteract cell proliferation in the overexpression group.
© 2000 Cancer Research Campaign

**Keywords:** human breast cancer; stathmin; quantitative RT-PCR; protein phosphorylation; prognostic factors; DNA

Stathmin (Sobel et al, 1989), also referred to as metablastin (Schubart et al, 1992), p19 (Pasmantier et al, 1986), prosolin (Cooper et al, 1989), p18 (Hanash et al, 1988), pp20 (Peyron et al, 1989) and Op18 (Hailat et al, 1990), is a small ubiquitous cytosolic phosphoprotein, proposed to be a relay integrating diverse cell signalling pathways (Sobel, 1991), as it is phosphorylated in parallel to the triggering of intracellular molecular cascades by hormones (Beretta et al, 1988, 1989; Sobel and Tashjian, 1983), growth factors (Doye et al, 1990) and neurotransmitters (Chneiweiss et al, 1992), and because it interacts with putative downstream protein partners including tubulin (Maucuer et al, 1995, 1997; Belmont and Mitchison, 1996; Curmi et al., 1997). A great deal of progress has recently been made in understanding the functions of stathmin, and it now appears that it may be one of the key regulators of the cell division through its influence on microtubule dynamics (Belmont and Mitchison, 1996; Marklund et al, 1996; Melander Gradin et al, 1997). Stathmin interacts with and sequesters free tubulin (Curmi et al, 1997; Jourdain et al, 1997) leading to the depolymerization of microtubules both in vitro and in vivo. Furthermore, phosphorylation of stathmin dramatically reduces its affinity for tubulin and thus its microtubule depolymerization potential (Marklund et al, 1996; Horwitz et al, 1997; Melander Gradin et al, 1997, 1998; Curmi et al, 1997; Di Paolo et al, 1997; Jourdain et al, 1997; Larsson et al, 1997; Gavet et al, 1998). The modulation of stathmin activity towards microtubules by phosphorylation is of the uttermost interest as it is known that stathmin is sequentially phosphorylated during the cell cycle, presumably through the concerted activity of different kinases, leading to an extensive phosphorylation of stathmin during mitosis (Strahler et al, 1992; Brattsand et al, 1994; Luo et al, 1994; Gavet et al, 1998) followed by a rapid dephosphorylation at cytokinesis (Gavet et al, 1998). Furthermore, the extensive phosphorylation of stathmin at mitosis seems to be required for cell cycle progression, as suggested by the cell cycle blockade at G2/M observed after cell transfection with various non-phosphorylatable stathmin mutants (Marklund et al, 1994; Larsson et al, 1995; Lawler et al, 1998).

With all these properties, stathmin appears thus as a good candidate for being implicated in cellular transformation or in cancer. In fact, we recently established that stathmin was overexpressed, at the mRNA and protein levels, in a significant proportion of human breast cancer that may define a new breast cancer subtype (Bièche et al, 1998). Overexpression of stathmin has also been reported in other types of malignancies such as acute leukaemia (Hanash et al, 1988; Brattsand et al, 1993; Ghosh et al, 1993; Luo et al, 1994; Melhem et al, 1997), lymphomas (Brattsand et al, 1993; Ghosh et al, 1993; Nylander et al, 1995), various carcinomas (Ghosh et al, 1993) and neuroblastomas (Hailat et al, 1990).

To this day, however, the significance of stathmin overexpression in cancer remains unclear. In particular, association with the standard prognostic markers has either not been searched for, or when studied no correlation has been really established mainly due to the rather small size of the series studied.

The aim of the present work was therefore to progress in the understanding of the significance and consequences of stathmin overexpression in cancer. We quantified by competitive reverse

*Received 10 March 1999*
*Revised 22 June 1999*
*Accepted 7 July 1999*

*Correspondence to:* R Lidereau

**Table 1** Distribution of clinical and biological parameters in a series of 133 patients with breast carcinoma. Relation to disease-free survival

| Variable | n (%) | Disease-free survival: Number of events[a] | P-value[b] |
|---|---|---|---|
| Age (years) | | | |
| ≤50 | 43 (32) | 13 | NS |
| >50 | 90 (68) | 35 | |
| Menopausal status | | | |
| Premenopausal | 49 (37) | 17 | NS |
| Post-menopausal | 84 (63) | 31 | |
| Histological grade[c,d] | | | |
| I | 18 (15) | 5 | |
| II | 61 (49) | 26 | NS |
| III | 45 (36) | 16 | |
| Nodal status | | | |
| 0 | 48 (36) | 9 | |
| 1–3 | 54 (41) | 25 | $P < 0.05$ |
| >3 | 31 (23) | 14 | |
| ER status | | | |
| + | 90 (68) | 35 | NS |
| – | 43 (32) | 13 | |
| PR status | | | |
| + | 79 (59) | 29 | NS |
| – | 54 (41) | 19 | |
| Tumour size[e] | | | |
| ≤ 30 mm | 93 (74) | 32 | NS |
| > 30 mm | 33 (26) | 14 | |

[a]First relapses (local and/or regional recurrences, and/or metastases), or breast cancer-related death without apparent relapses. [b]Log-rank test. [c]Scarff Bloom Richardson classification. [d]Information available for 124 patients. [e]Information available for 126 patients. ER, oestrogen receptors; PR, progesterone receptors (cut-off for ER and PR, 10 fm mg$^{-1}$); *n*: number of patients; NS: not significant.

transcriptase polymerase chain reaction (RT-PCR) the amount of stathmin mRNA in a large series ($n$ = 133) of unilateral invasive primary breast tumour samples, obtained in strictly followed up women whose clinico-pathological data were fully available. We then searched for potential correlation of stathmin overexpression with standard prognostic factors, and examined whether alterations in stathmin expression is associated with loss of heterozygosity (LOH) at the stathmin locus.

### MATERIALS AND METHODS

### Patients' characteristics and samples

We analysed tissue from excised primary breast tumour samples from a series of 133 women, collected between 1977 and 1989 at the Centre René Huguenin (St-Cloud, France). The samples were first examined histologically for the presence of tumour cells, a tumour sample being considered suitable for further analysis if the proportion of tumour cells was more than 60%. Immediately after surgical removal, the tumour samples were snap-frozen and stored in liquid nitrogen until the time of extraction of RNA and high-molecular-weight DNA.

Peripheral blood from the same patients was also obtained and leucocytes were used as a source of patient's normal DNA for comparison.

Patient and tumour details included in the present study are listed in Table 1, showing these to be a typically representative cohort of breast cancer patients. Patients met the following criteria: primary unilateral non-metastatic breast carcinoma; the mean age was 57 years (range 34–86); complete clinical, histological and biological data; no radiotherapy or chemotherapy before surgery. The median follow-up of these patients was long, i.e. 9 years (range 1.0–16.2). Forty-eight patients relapsed during the follow-up period (15 local and/or regional recurrences, 29 metastases and four patients with both were the first relapse events). Two second invasive cancers occurred (not considered as relapse events).

The control RNAs assayed were from seven uninvolved breast tissue adjacent to carcinoma, and eight normal breast tissue collected from reduction mammoplasties. To these, we added RNA derived from a pool of six normal human breast tissues purchased at Clontech (Cat. no. 64037–1). All these RNAs are referred to as normal.

### Evaluation of 'classical' prognostic factors

Histological typing was established at the time of surgery according to the Bloom and Richardson classification (Bloom and Richardson, 1957). Oestrogen and progesterone receptors content of the tumours was assayed according to the guidelines of the European Organisation for Research and Treatment for Cancer (EORTC Breast Cooperative Group, 1980), with a detection threshold of 10 fmol mg$^{-1}$ cytosolic protein.

### RNA analysis

#### *RNA extraction and cDNA synthesis*

Total RNA was extracted from pulverized frozen breast tissues by using the acid–guanidinium thiocyanate–phenol–chloroform method (Chomczynski and Sacchi, 1987). Quality and purity of RNA were assessed by electrophoresis on 1.2% agarose gels. Efficiency of recovery was calculated on an aliquot by determining the A260.

Reverse transcription was performed on 1 μg of total RNA in a final volume of 20 μl. The reaction tube contained 2.5 mM random hexamers, 20 units of RNAase inhibitor, and 50 units of Moloney Murine Leukaemia Virus reverse transcriptase (Perkin-Elmer Applied Biosystems) in a 1× RT-PCR buffer (1 mM each dNTP, 5 mM magnesium chloride ($MgCl_2$) 50 mM potassium chloride (KCl), 10 mM Tris–HCl pH 8.3 at 25°C). After 10 min of incubation at 20°C, and 30 min at 42°C, the reverse transcriptase was inactivated by heating at 99°C for 5 min, and the cDNA was quickly chilled on ice to 5°C. The volume of the reaction mixture was then brought to 200 μl with 1× RT-PCR buffer, giving the 'RT-mix' used for subsequent PCR.

#### *PCR primers*

Primer pairs for PCR were designed with the assistance of the Oligo 4.0 computer program (National Biosciences, Plymouth, MN, USA) and selected after performing BLASTN searches against nr (All Non-redundant GenBank+EMBL+DDBJ+PDB sequences (but no EST, STS, GSS, or HTGS sequences)) and dbEST to confirm the total gene specificity of the primer sequences and the absence of polymorphism. Primers are designated by the nucleotide position corresponding to the 5′ position (relative to stathmin GenBank no. X53305 and TBP GenBank no. X54993), with the letter U for upper (sense strand) or L for lower (antisense strand). Primers were 6U: 5′ F-CTCGGACT-GAGCAGGACTTTC 3′ and 172L: 5′ ATTCTTTTGACC-

© 2000 Cancer Research Campaign

GAGGGCTG 3′ for the stathmin gene; and 750U: 5′ F-ACA-GGAGCCAAGAGTGAAGAA 3′ and 1009L: 5′ CCAGAAA-CAAAAATAAGGAGA 3′ for the TBP gene (F: fluorescein molecule). These primers gave a PCR product of 167 bp after 30 PCR cycles for the stathmin gene and 260 bp after 32 PCR cycles for the TBP gene. To avoid amplification of contaminating genomic DNA, one of the two primers was chosen at the junction between two exons or in a different exon. The upper primer of stathmin (6U) thus spanned the junction between exons 1 and 2, whereas the lower primer (172L) was placed in exon 3. One member of each primer pair was fluorescein-labelled at the 5′ end with fluorescein by the fluoro-prime method.

### *Quantitative analysis of stathmin mRNA by RT-PCR*

Stathmin mRNA expression was quantified by RT-PCR using the TBP transcript (TATA box Binding Protein, a major component of the TFIID transcription factor) as an endogenous mRNA control, and each sample was normalized on the basis of its TBP content. TBP was chosen because no retro-pseudogenes have been found for it (the existence of retro-pseudogenes leads to co-amplification of contaminating genomic DNA despite the use of primers from separate exons) and because it shows an approximately similar abundance of transcripts to that of stathmin.

To take into account the variability of PCR efficiency, we co-amplified, using the same pair of primers, target cDNAs (stathmin or TBP) with a ‘quantitative DNA standard’, QDS (Foley et al, 1993). QDS for each target gene contained a 12 bp insertion, and was generated by PCR using the ‘looped oligo’ method (Sarkar and Bolander, 1994) as described by Lazar et al (1995). Target cDNAs and QDS thus yielded labelled PCR products of different sizes which were easily identified after migration on a DNA sequencing gel. The ratio between amplified target cDNA and its QDS was checked to be constant during exponential and non-exponential phase of PCR, according to results of Pannetier et al (1993). Gene mRNA amount was expressed as the ratio between target cDNA and its corresponding QDS.

For each PCR reaction, a master mix was prepared on ice with 1.5 mM $MgCl_2$, 50 mM KCl, 10 mM Tris–HCl pH 8.3 at 25°C, 400 nM of each primer, a known amount of the corresponding QDS and 1 unit of AmpliTaq DNA polymerase (Perkin-Elmer Applied Biosystems). In the control group, QDS amount was adjusted in order to obtain similar fluorescence intensities for QDS and the corresponding cDNA-derived PCR products. Ten microlitres of RT-mix for each sample were added on ice to 40 μl of the master mix. PCR consisted of an initial denaturation at 94°C for 5 min and 30–32 cycles of 30 s at 94°C, 30 s at 60°C, 45 s at 72°C, and a final extension of 10 min at 72°C, using a Perkin-Elmer 9600 DNA thermocycler.

PCR products were diluted tenfold and 2.5 μl diluted aliquots of each sample were added to 2.5 μl of deionized formamide containing 0.3 μl of molecular size markers (Genescan 500 ROX, Perkin-Elmer Applied Biosystems). The mixtures were heat-denatured and 2.5 μl aliquots were loaded on 6% polyacrylamide/8 M urea gel and run for 6 h at 1200 V in a sequencer. The amount of the different fragments was quantified with the Genescan® 672 Fragment Analysis software (Perkin-Elmer Applied Biosystems). Areas of each fluorescent peak was expressed as relative fluorescence units (RFU), which correlates to the amount of PCR product.

Quantification was based on the determination of the relative amount of the stathmin target message to the TBP endogenous control to normalize the amount and quality of total RNA. Final results, expressed as N-fold differences in stathmin gene expression relative to TBP gene expression, termed ‘Stathmin (normalized)’, were determined as follows:

$$\text{Stathmin (normalized)} = [\text{stathmin}/\text{stathmin}_{\text{QDS}}]/[\text{TBP}/\text{TBP}_{\text{QDS}}]$$

All the results of the present work regarding stathmin mRNA expression are therefore the stathmin normalized value. Experiments were performed with duplicates for each data point and the mean value was determined. Reproducibility of the quantitative measurements was evaluated by conducting independent replicate cDNA syntheses and PCR reactions.

### DNA analysis

DNA was extracted from frozen tumour tissue and blood leucocytes from each patient, by using standard methods (Sambrook et al, 1989).

In order to identify all patients informative for at least one locus, the carcinomas were screened with three polymorphic microsatellite DNA marker loci flanking stathmin (D1S234, D1S1676, D1S2734). Normal DNA samples which were polymorphic at a given locus were considered to be ‘informative’, whereas homozygous were considered ‘uninformative’. Only cases of constitutional heterozygosity were used in the evaluation of LOH.

Microsatellite markers were assayed by PCR amplification of genomic DNA. PCR was run in a total volume of 50 μl, with 50 ng of genomic DNA, 400 nM each primer, 200 μM of each dNTP, and one unit of AmpliTaq DNA polymerase (Perkin-Elmer Applied Biosystems) (in 1.5 mM $MgCl_2$, 50 mM KCl, 10 mM Tris–HCl pH 8.3 at 25°C). The annealing temperature, number of amplification cycles and extension time were adapted for each primer set. One microlitre of product was mixed with 3 μl of denaturing loading buffer, heat-denatured, then 1.5 μl aliquots of each sample were loaded on 6% polyacrylamide/7.5 M urea gels. After fractionation, DNA was transferred to nylon membrane filters. The membrane filters were hybridized overnight at 42°C with the CA repeat $^{32}$P-labelled probe, washed, and autoradiographed at –80°C for an appropriate period.

The signal intensity of the polymorphic alleles was evaluated by independent visual examination by three observers and confirmed by means of densitometry. The results of all the scanned samples were in direct agreement with the initial visual scoring. LOH was considered to occur when the intensity of the allele in tumour DNA was less than 40% of that measured in the corresponding normal DNA (peripheral blood lymphocytes). LOH was partial in most cases, the band being fainter than the conserved allele but still visible. Such partial losses are due either to contaminating normal tissue or to tumour heterogeneity.

### Statistical analysis

Disease-free survival (DFS) was determined as the interval between diagnosis and detection of the first relapses (local and/or regional recurrences, and/or metastases), or breast cancer-related death without apparent relapses.

Clinical, histological and biological parameters were compared using the $\chi^2$ test. Differences between the two populations were judged significant at confidence levels greater than 95% ($P < 0.05$). Survival distributions were estimated by the Kaplan–Meier method (Kaplan and Meier, 1958), and the

*© 2000 Cancer Research Campaign*

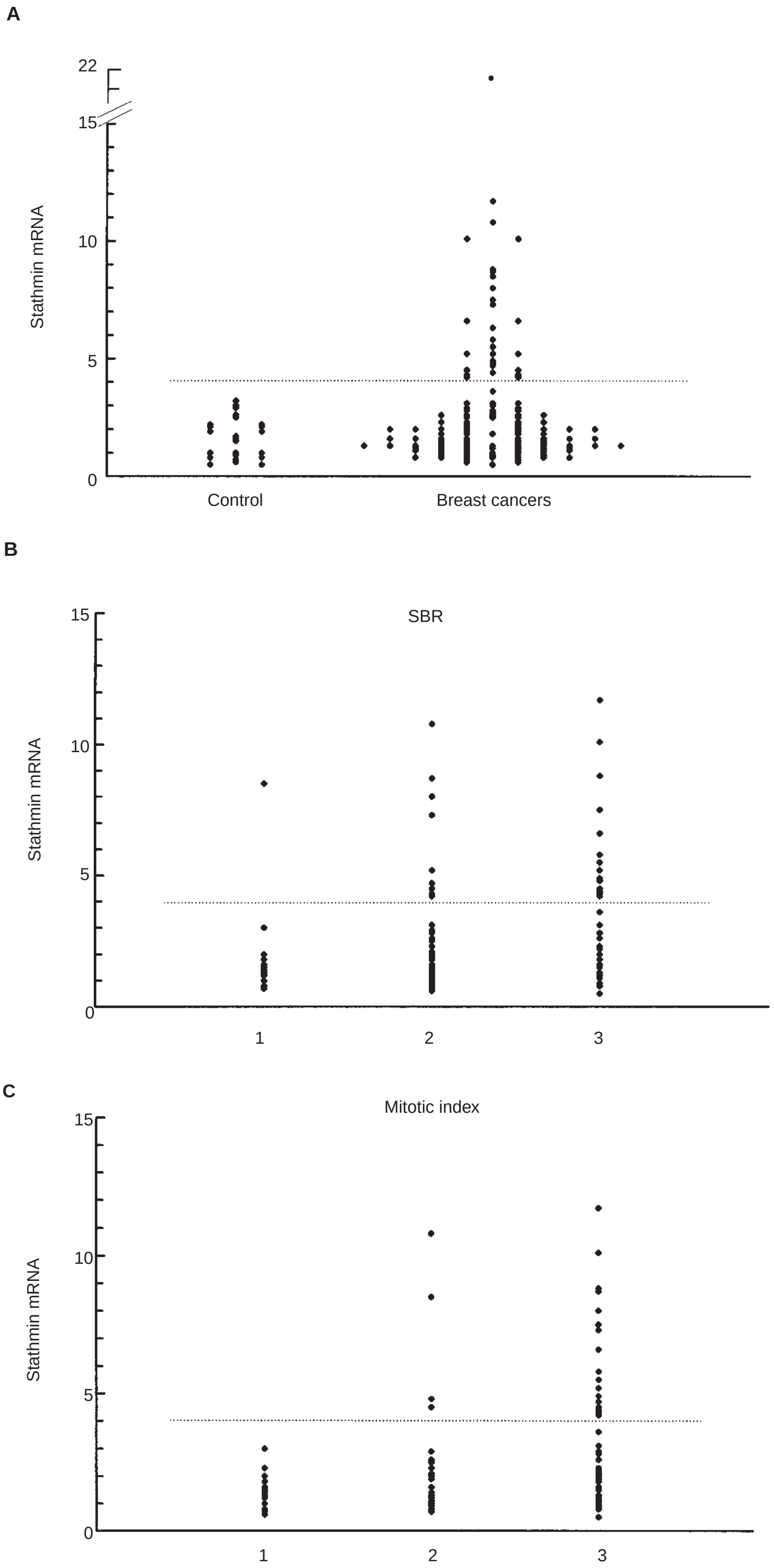

**Figure 1** Expression of stathmin mRNA in control tissues and breast carcinomas, quantified by RT-PCR. **(A)** Scattergram showing stathmin expression obtained from either normal breast RNA or breast cancer biopsies as described in Patients and Methods. Stathmin expression in control samples varied between 0.5 and 3.1 (mean 1.63 ± 0.84). The stathmin overexpression threshold was calculated to 4. In the cancer sample an overexpressing subgroup of 29 tumours (21.8%) was clearly distinguishable (range 4.2–21.7; mean 7.04 ± 3.58). **(B)** Scattergram showing stathmin expression in breast cancer and its distribution in the three SBR grading groups. A significant correlation was found between stathmin expression and grade III disease ($P = 0.002$). **(C)** Scattergram showing stathmin expression in breast cancer and its distribution for the three mitotic index scores. A significant correlation was found between stathmin expression and score 3 of the mitotic index ($P = 0.02$). *Information was not available for the tumour with a 21.7 stathmin expression level reported in (**A**)

*© 2000 Cancer Research Campaign* 

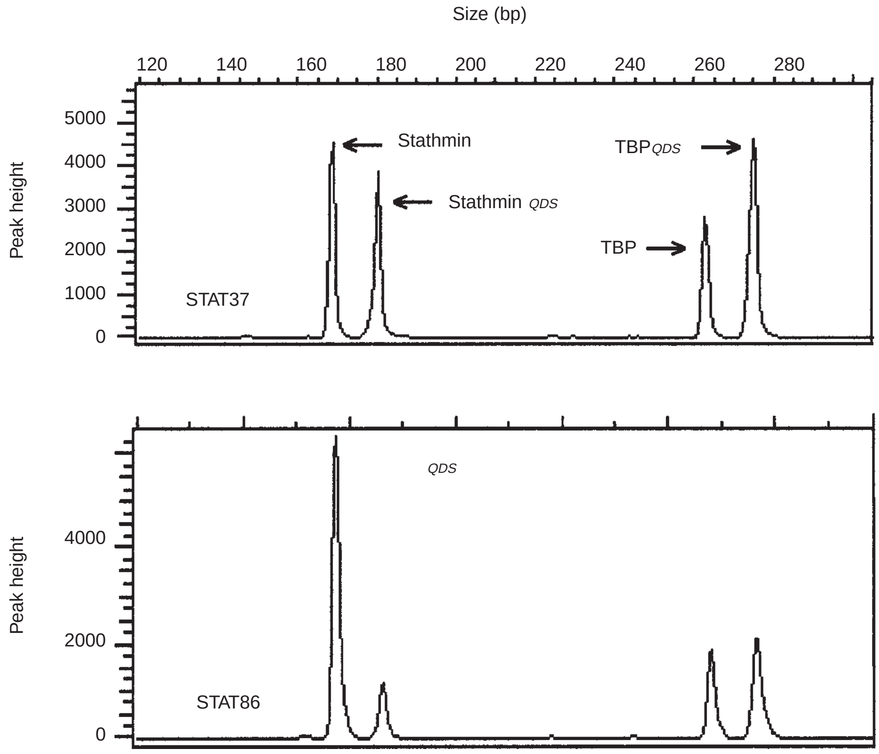

| Sample | Strathmin | | | TBP | | | Stathmin (normalized) |
|---|---|---|---|---|---|---|---|
| | Sta | Sta*QDS* | Sta/Sta | TBP | TBP*QDS* | TBP/TBP*QDS* | |
| STAT37 | 33482 | 29579 | 1.13 | 19337 | 37915 | 0.51 | 2.2 |
| STAT86 | 53670 | 10361 | 5.18 | 16328 | 22264 | 0.73 | 7.1 |

**Figure 2** RT-PCR analysis of the stathmin mRNA content of two breast tumour samples. Peaks stathmin (or TBP) and stathmin$_{QDS}$ (or TBP$_{QDS}$) represent the PCR products of the stathmin cDNA (or TBP) and of its corresponding QDS. The area of each peak correlates with the amount of PCR product. For each tumour sample, the stathmin cDNA peak area value (Sta) was divided by the stathmin QDS peak area value (StaQDS) to obtain a stathmin mRNA amount value (Sta/StaQDS) which was next divided by the TBP mRNA amount value (TBP/TBPQDS) to obtain the final stathmin (normalized) value. Duplicate analysis for each tumour sample were performed (a single is shown here). The stathmin (normalized) value was equal to 2.2 in tumour STAT no. 37 and 7.1 in tumour STA no. 86

significance of differences between survival rates was ascertained using the log-rank test (Peto et al, 1977).

### RESULTS

In order to assess the significance of stathmin overexpression in breast cancer, we evaluated the correlation of stathmin expression, quantified by means of RT-PCR analysis, with several disease parameters in a large series of human primary breast cancer obtained in strictly followed up women whose clinico-pathological data were fully available (Table 1).

### Expression of stathmin mRNA in breast tissues

Stathmin mRNA expression in normal ($n$ = 16) or breast cancer specimens ($n$ = 133) was determined by RT-PCR. The results were standardized by comparison with the mRNA level of an endogenous mRNA control to give the stathmin mRNA normalized value (see Patients and Methods). The stathmin mRNA expression values reported in this paper are therefore the normalized stathmin mRNA values.

Stathmin mRNA expression was first assessed for the control normal breast RNAs. Expression levels varied between 0.5 and 3.1 (mean 1.63 ± s.d. 0.84) (Figure 1A). From these values, we determined the cut-off for altered stathmin mRNA expression in breast cancer as the mean value ± 3 s.d. observed for the control normal breast mRNA. Stathmin mRNA was thus considered to be overexpressed for expression values ≥ 4.

Stathmin mRNA expression was next quantified in the breast cancer samples ($n$ = 133). Levels of stathmin varied between 0.5 and 21.7 (Figure 1A). Using the stathmin overexpression cut-off value of 4, we classified tumours in two stathmin expression groups, basal and overexpression. In the basal group ($n$ = 104), the mean value of stathmin mRNA expression was equal to 1.65 ± 0.7 (range 0.5–3.8) a value not distinguishable from that found in the control normal group. In the overexpression group ($n$ = 29) the mean value of stathmin mRNA expression was equal to 7.04 ± 3.58 (range 4.2–21.7). Stathmin expression thus varied in this group between 2.6 and 13.3 times the mean control value. An example of tumours in which the expression level was basal (STAT no. 37, stathmin = 2.2) and high (STAT no. 86, stathmin = 7.1) is presented in Figure 2.

 *© 2000 Cancer Research Campaign*

**Table 2** Association between stathmin mRNA expression and the different prognostic criteria

| | | Stathmin mRNA | | |
|---|---|---|---|---|
| Variable | *n* (%) | Basal *n* (%) | Overexpression *n* (%) | *P*-value[a] |
| Total | 133 (100) | 104 (78) | 29 (22) | |
| Age (years) | | | | |
| ≤50 | 43 (32) | 34 (33) | 9 (31) | NS |
| >50 | 90 (68) | 70 (67) | 20 (69) | |
| Menopausal status | | | | |
| Premenopausal | 49 (37) | 39 (37) | 10 (34) | NS |
| Post-menopausal | 84 (63) | 65 (63) | 19 (66) | |
| Histological grade[b,c] | | | | |
| I | 18 (15) | 17 (17) | 1 (4) | 0.002 |
| II | 61 (49) | 53 (54) | 8 (31) | |
| III | 45 (36) | 28 (29) | 17 (65) | |
| Nodal status | | | | |
| 0 | 48 (36) | 34 (33) | 14 (48) | NS |
| 1–3 | 54 (41) | 45 (43) | 9 (31) | |
| >4 | 31 (23) | 25 (24) | 6 (21) | |
| ER status | | | | |
| + | 90 (68) | 78 (75) | 12 (41) | 0.0006 |
| – | 43 (32) | 26 (25) | 17 (59) | |
| PR status | | | | |
| + | 79 (59) | 68 (65) | 11 (38) | 0.008 |
| – | 54 (41) | 36 (35) | 18 (62) | |
| Tumour size[d] | | | | |
| ≤30 mm | 93 (74) | 74 (75) | 19 (70) | NS |
| >30 mm | 33 (26) | 25 (25) | 8 (30) | |
| Relapses | | | | |
| + | 48 (36) | 40 (38) | 8 (28) | NS |
| – | 85 (64) | 64 (62) | 21 (72) | |

[a]Chi-square test. [b]Scarff–Bloom–Richardson classification. [c]Information available for 124 patients. [d]Information available for 126 patients. ER, oestrogen receptors; PR, progesterone receptors (cut-off for ER and PR, 10 fm mg$^{-1}$); *n*: number of patients; NS: not significant

**Table 3** Association between stathmin mRNA expression and the SBR histopathological grade components

| | | Stathmin mRNA | | |
|---|---|---|---|---|
| | *n* (%) | Basal *n* (%) | Overexpression *n* (%) | *P*-value[a] |
| Total | 124 (100) | 98 (79) | 26 (21) | |
| Tubular differentiation | | | | |
| I | 5 (4) | 4 (4) | 1 (4) | |
| II | 80 (65) | 68 (69) | 12 (46) | NS |
| III | 39 (31) | 26 (27) | 13 (50) | |
| Nuclear polymorphism | | | | |
| I | 7 (6) | 7 (7) | 0 | |
| II | 84 (68) | 68 (69) | 16 (62) | NS |
| III | 33 (26) | 23 (24) | 10 (38) | |
| Mitotic index | | | | |
| I | 16 (13) | 16 (16) | 0 | |
| II | 30 (24) | 26 (27) | 4 (15) | 0.02 |
| III | 78 (63) | 56 (57) | 22 (85) | |

[a]Chi-square test with Yates' correction. *n*: number of patients, NS: not significant.

**Table 4** Association between stathmin DNA status and stathmin mRNA expression[a]

| | | | Stathmin mRNA | | |
|---|---|---|---|---|---|
| | | Total population *n* (%) | Basal *n* (%) | Overexpression *n* (%) | |
| 1p36 | Yes | 33(29) | 24(28) | 9(33) | |
| LOH | No | 79(71) | 61(72) | 18(67) | NS |

[a]Frequency of chromosome 1p36.1 alteration as determined with the D1S234, D1S1676 and D1S2734 markers: alteration in breast tumours and association with stathmin mRNA overexpression. *n*: number of patients, NS: not significant.

### Correlation of stathmin mRNA expression in breast cancers with standard prognostic factors

We sought links between stathmin mRNA level status and standard clinico-pathological and biological factors in breast cancer (Table 2). Interestingly, stathmin overexpression was significantly correlated with a number of parameters known to be associated with poor prognosis. Overexpression was correlated with grade III disease ($P$ = 0.002) (Table 2 and Figure 1B), loss of oestrogen receptor ($P$ = 0.0006) or progesterone receptor ($P$ = 0.008). In the SBR grading, three morphological features (tubular formation, nuclear pleomorphism and mitotic index) were scored from 1 to 3. When we tested stathmin mRNA expression with regard to these three components (Table 3), it appears that the association of stathmin overexpression with SBR grade III was essentially due to the mitotic index ($P$ = 0.02) (Figure 1B, C).

These results indicate that overexpression of stathmin is associated with highly proliferative primary breast carcinoma showing signs of aggressiveness. However, it is interesting to note that no association was observed with axillary lymph node. Moreover, patients with tumours overexpressing stathmin did not relapse more frequently (Table 2) and did not have significantly shorter disease-free survival after surgery compared to patients with tumours showing basal level of stathmin expression (log-rank test).

### Relationship between the stathmin mRNA levels and 1p36.1 LOH

Assessment of LOH (loss of heterozygosity) with three polymorphic DNA markers (D1S234, D1S1676, D1S2734) encompassing 1 cM in 1p36.1 and flanking the stathmin gene (Jensen et al, 1997) is shown in Table 4. 116 of the 133 tumours studied for stathmin expression were tested for LOH. Of these, 112 patients were informative for one or more loci on 1p36.1. LOH was found in 29.5% (33/112) of the informative tumour DNAs, while the remainder had a normal profile. As shown in Table 4, we found no link between stathmin LOH and stathmin mRNA overexpression indicating that deletion of the stathmin gene or of the region encompassing it may not be the cause of its overexpression.

### DISCUSSION

We recently demonstrated that stathmin was overexpressed, at the mRNA and protein levels, in a significant proportion of human breast cancers (about 30%) that may define a new breast cancer subtype (Bièche et al, 1998). In this pilot study, we found essentially no significant correlation between stathmin overexpression and any of the classical prognostic parameters because the sample size ($n$ = 50) was too small to attempt reasonable statistical correlation. The aim of the present work was thus to evaluate the prognostic significance of stathmin overexpression, quantified with a competitive RT-PCR-based strategy, in a larger population of human breast cancer ($n$ = 133) for which clinico-pathological data were fully available. Our analyses of stathmin expression were restricted to mRNA because we showed previously that a perfect match existed, in breast cancer, between stathmin mRNA and protein expression (appreciated by Western blotting and immunohistochemistry), suggesting that no post-transcriptional regulation occurred for stathmin.

First of all our results allowed us to refine the data obtained in our initial survey. We definitely confirmed the existence of two statistically different populations (or groups) of breast cancers in view of stathmin expression: a basal group whose expression level was indistinguishable from non tumoural breast samples considered as control, and an overexpressing group in which stathmin expression levels were quite higher. The level of stathmin mRNA in the overexpression group varied between 2.6 and 13.3 times (mean 4.26) that found in the basal group. This result is in good agreement with the mRNA values estimated by means of Northern blot in our initial work. With our new data set, the percentage of breast cancers that overexpress stathmin was adjusted to about 22%, a value again not far from the 30% reported in the previous study, but which reflects with a better accuracy the real extent of this population. Finally our present analysis allows to restrict the hypotheses about the mechanism(s) of stathmin overexpression in view of the lack of actually significant correlation between stathmin overexpression and 1p36.1 LOH. This result clearly rules out the possibility that overexpression may be related to a deletion in the region of the stathmin gene.

Analysis of the data for association between stathmin overexpression and a number of disease parameters revealed that stathmin overexpression is significantly correlated to some parameters known to be associated with poor prognosis. Overexpression is correlated with grade III disease, and loss of oestrogen or progesterone receptors. While the SBR grade III correlation is of interest, we show further that it is due to a significant relationship between overexpression of stathmin and the score 3 of the mitotic index, one of the three components of the SBR grading. Overexpression of stathmin thus amazingly points out to highly proliferative primary breast carcinomas showing signs of aggressiveness, although no association was observed with axillary lymph node and stathmin mRNA overexpression is not a predictor for a shorter disease-free survival.

Stathmin overexpression has already been observed in different types of cancers but, except for the work of Friedrich et al conducted on a limited number of human prostatic carcinomas ($n$ = 40) (Friedrich et al, 1995), has not been correlated to human clinico-pathological parameters. Thus, to the best of our knowledge, our work is the first that reports an evaluation of stathmin expression in such a large series of human cancer allowing clear statistical analysis. Our results raise questions that need to be clarified:

1. Does stathmin overexpression contribute to tumorigenesis? Experimental data from our laboratory (Koppel et al, 1993; Balogh et al, 1996) indicate that stathmin expression is up-regulated at a stage that precedes cell differentiation in relation to the limitation of cell overgrowth. We could thus envision that overexpression of stathmin may be part of a regulatory loop aimed at counteracting cell proliferation, although inefficiently in cancer cells.
2. What are the mechanisms which may have led to stathmin overexpression? Our data indicate that stathmin overexpression may be related to an increased transcription or to a messenger stabilization. The analysis of the 5′-flanking sequences of the stathmin gene (Melhem et al, 1991) has revealed the presence of several transcription factor recognition sequences: two AP-2 sites, five GC boxes which fit the Sp1 consensus recognition sequences and four E2F sites. AP 2 family may participate to the well known overexpression of the proto-oncogene c-*erb*B 2 observed in about 25–30% of the breast cancers (Slamon et al, 1989). In parallel to the present study, c-*erb*B-2 was quantified by RT-PCR in our series of tumours (Bièche et al, 1999) and no association was noted between stathmin and c-*erb*B-2 overexpression. Sp1 recognizes GC box sequences in a number of cellular and viral promoters (Kadonaga et al, 1997), and is essential for transcription from TATA-less promoters (Pugh and Tjian, 1990, 1991) which is the case of the stathmin gene (Melhem et al, 1991). An increase in Sp1 transcription or a stabilization of the Sp1 protein by a post-translational modification such as glycosylation (Han and Kudlow, 1997) could thus be conceivable for being the cause of a stathmin over-transcription. On the other hand, E2F is probably not involved since it has been shown that overexpression of members of the E2F family induced apoptosis in human breast cancer cell lines (Hunt et al, 1997) while its blocking, in contrast, inhibits apoptosis (Bargou et al, 1996).
3. Another point raised by our work is to understand the reasons of the apparent inefficiency of stathmin to block or to slow down the cell cycle. Knowing the dramatic effects of stathmin toward microtubule depolymerization in vitro (Belmont and Mitchison, 1996; Horwitz et al, 1997; Jourdain et al, 1997) and in vivo (Gavet et al, 1998), as well as its ability to counteract the cell cycle (Marklund et al, 1994; Larsson et al, 1995; Lawler et al, 1998), the coexistence of a high mitotic index with stathmin overexpression is particularly amazing. Different hypotheses can be proposed to account for this apparent contradiction: (i) a novel equilibrium is reached between stathmin and tubulin due to a compensatory increase in the overall cellular content of tubulin; (ii) the stathmin–tubulin interaction is altered. However the stathmin side seems not to be affected since the protein migrates at the right place on two-dimensional PAGE (Hailat et al, 1990; Bièche et al, 1998); we thus cannot exclude that some sort of tubulin alteration or third partner could impair the stathmin–tubulin interaction; (iii) stathmin is massively phosphorylated and thus rendered inactive in mitosis. Data already published on stathmin phosphorylation in tumours, including ours, indicate that apparently it is poorly phosphorylated.

In conclusion, we demonstrated that stathmin is overexpressed, by increased transcription or mRNA stabilization, in about 22% of human primary breast tumours and points out to highly proliferative primary breast carcinomas showing signs of aggressiveness. Our results are in an apparent contradiction with the current knowledge of the stathmin anti-proliferative potential and raise questions regarding the causes of its overexpression and, more importantly, the reasons of its inability to counteract cellular proliferation. The existence of a stathmin overexpressing group in

*© 2000 Cancer Research Campaign*

human breast carcinomas offers a new target for drug or gene therapy directed toward the disturbance of the stathmin–tubulin equilibrium which should block the cell cycle, as data obtained from cellular transfections with antisense (Luo et al, 1994) or sense non-phosphorylatable stathmin (Marklund et al, 1994; Gavet et al, 1998; Lawler et al, 1998) indicate that any acute change of the stathmin–tubulin equilibrium should lead to a substantial cell cycle inhibition.

### ACKNOWLEDGEMENTS

This work was supported by funds from the Association pour la Recherche contre le Cancer (ARC), the Association Française contre les Myopathies (AFM), the Institut National de la Santé et de la Recherche Médicale (INSERM) and the Ligue Nationale Contre le Cancer (LNCC). We thank A Khodja for excellent technical assistance. We thank the Centre René Huguenin staff for assistance in specimen collection and patient care.

### REFERENCES

Balogh A, Mège RM and Sobel A (1996) Growth and cell density-dependent expression of stathmin in C2 myoblasts in culture. *Exp Cell Res* **224**: 8–15

Bargou RC, Wagener C, Bommert K, Arnold W, Daniel PT, Mapara MY, Grinstein E, Royer HD and Dorken B (1996) Blocking the transcription factor E2F/DP by dominant-negative mutants in a normal breast epithelial cell line efficiently inhibits apoptosis and induces tumor growth in SCID mice. *J Exp Med* **183**: 1205–1213

Belmont LD and Mitchison TJ (1996) Identification of a protein that interacts with tubulin dimers and increases the catastrophe rate of microtubules. *Cell* **84**: 623–631

Beretta L, Boutterin MC and Sobel A (1988) Phosphorylation of intracellular proteins related to the multihormonal regulation of prolactin: comparison of normal anterior pituitary cells in culture with the tumor-derived GH cell lines. *Endocrinology* **122**: 40–51

Beretta L, Boutterin MC, Drouva S and Sobel A (1989) Phosphorylation of a group of proteins related to the physiological, multihormonal regulations of the various cell types in the anterior pituitary gland. *Endocrinology* **125**: 1358–1364

Bièche I, Lachkar S, Becette V, Cifuentes-Diaz C, Sobel A, Lidereau R and Curmi P (1998) Overexpression of the stathmin gene in a subset of human breast cancer. *Br J Cancer* **78**: 701–709

Bièche I, Onody xP, Laurendeau I, Olivi M, Vidaud, Lidereau R and Vidaud M (1999) Real-time reverse transcription-PCR assay for future management of ERBB2-based clinical applications. *Clin Chem* **45**: 1148–1156

Bloom HJG and Richardson WW (1957) Histological grading and prognosis in breast cancer. *Br J Cancer* **11**: 359–377

Brattsand G, Roos G, Marklund U, Ueda H, Landberg G, Nanberg E, Sideras P and Gullberg M (1993) Quantitative analysis of the expression and regulation of an activation-regulated phosphoprotein (oncoprotein 18) in normal and neoplastic cells. *Leukemia* **7**: 569–579

Brattsand G, Marklund U, Nylander K, Roos G and Gullberg M (1994) Cell-cycle-regulated phosphorylation of oncoprotein 18 on ser16, ser25, and ser38. *Eur J Biochem* **220**: 359–368

Chneiweiss H, Cordier J and Sobel A (1992) Stathmin phosphorylation is regulated in striatal neurons by vasoactive intestinal peptide and monoamines via multiple intracellular pathways. *J Neurochem* **58**: 282–289

Chomczynski P and Sacchi N (1987) Single-step method of RNA isolation by acid guanidinium thiocyanate-phenol-chloroform extraction. *Anal Biochem* **162**: 156–159

Cooper HL, McDuffie E and Braverman R (1989) Human peripheral lymphocyte growth regulation and response to phorbol esters is linked to synthesis and phosphorylation of the cytosolic protein, prosolin. *J Immunol* **143**: 956–963

Curmi PA, Andersen SSL, Lachkar S, Gavet O, Karsenti E, Knossow M and Sobel A (1997) The stathmin tubulin interaction in vitro. *J Biol Chem* **272**: 25029–25036

Di Paolo G, Antonsson B, Kassel D, Riederer BM and Grenningloh G (1997) Phosphorylation regulates the microtubule-destabilizing activity of stathmin and its interaction with tubulin. *FEBS Lett* **416**: 149–152

Doye V, Boutterin MC and Sobel A (1990) Phosphorylation of stathmin and other proteins related to nerve growth factor-induced regulation of PC12 cells. *J Biol Chem* **265**: 11650–11655

EORTC Breast Cooperative Group (1980) Revision of the standards for the assessment of hormone receptors in human breast cancer. Report of the second EORTC Workshop, held on 16–17 March 1979, in the Netherlands Cancer Institute. *Eur J Cancer* **16**: 1513–1516

Foley KP, Leonard MW and Engel JD (1993) Quantitation of RNA using the polymerase chain reaction. *TIG* **9**: 380–385

Friedrich B, Grönberg H, Landström M, Bergh A and Gullberg M (1995) Differentiation-stage specific expression of oncoprotein 18 in human and rat prostatic adenocarcinoma. *Prostate* **27**: 102–109

Gavet O, Ozon S, Manceau V, Lawler S, Curmi P and Sobel A (1998) The stathmin phosphoprotein family: intracellular localization and effects on the microtubule network. *J Cell Sci* **111**: 3333–3346

Ghosh PK, Anderson NG, Cohen P, Taketo M, Atweh GF and Lebowitz P (1993) Expression of the leukemia-associated gene p18 in normal and malignant tissues: inactivation of expression in a patient with cleaved B-cell lymphoma/leukemia. *Oncogene* **8**: 2869–2872

Hailat N, Strahler JR, Melhem RF, Zhu XX, Brodeur G, Seeger RC, Reynolds CP and Hanash SM (1990) N-myc gene amplification in neuroblastoma is associated with altered phosphorylation of a proliferation related polypeptide (Op 18). *Oncogene* **5**: 1615–1618

Han I and Kudlow JE (1997) Reduced O glycosylation of Sp1 is associated with increased proteasome susceptibility. *Mol Cell Biol* **17**: 2550–2558

Hanash SM, Strahler JR, Kuick R, Chu EHY and Nichols D (1988) Identification of a polypeptide associated with the malignant phenotype in the acute leukemia. *J Biol Chem* **263**: 12813–12815

Horwitz SB, Shen H-J, He L, Dittmar P, Neef R, Chen J and Schubart UK (1997) The microtubule-destabilizing activity of metablastin (p19) is controlled by phosphorylation. *J Biol Chem* **272**: 8129–8132

Hunt KK, Deng J, Liu TJ, Wilson-Heiner M, Swisher SG, Clayman G and Hung MC (1997) Adenovirus-mediated overexpression of the transcription factor E2F-1 induces apoptosis in human breast and ovarian carcinoma cell lines and does not require p53. *Cancer Res* **57**: 4722–4726

Jensen SJ, Sulman EP, Maris JM, Matise TC, Vojta PJ, Barrett JC, Brodeur GM and White PS (1997) An integrated transcript map of human chromosome 1p35–p36. *Genomics* **42**: 126–136

Jourdain L, Curmi P, Sobel A, Pantaloni D and Carlier MF (1997) Stathmin: a tubulin-sequestering protein which forms a ternary T2S complex with two tubulin molecules. *Biochemistry* **36**: 10817–10821

Kadonaga JT, Carner KR, Masiarz FR and Tjian R (1997) Isolation of cDNA encoding transcription factor Sp1 and functional analysis of the DNA binding domain. *Cell* **51**: 1079–1090

Kaplan EL and Meier P (1958) Nonparametric estimation from incomplete observations. *J Am Stat Assoc* **53**: 457–481

Koppel J, Loyer P, Maucuer A, Rehák P, Manceau V, Guguen-Guillouzo C and Sobel A (1993) Induction of stathmin expression during liver regeneration. *FEBS Lett* **331**: 65–70

Larsson N, Melander H, Marklund U, Osterman O and Gullberg M (1995) G2/M transition requires multisite phosphorylation of oncoprotein 18 by two distinct protein kinase systems. *J Biol Chem* **270**: 14175–14183

Larsson N, Marklund U, Gradin HM, Brattsand G and Gullberg M (1997) Control of microtubule dynamics by oncoprotein 18: dissection of the regulatory role of multisite phosphorylation during mitosis. *Mol Cell Biol* **17**: 5530–5539

Lawler S, Gavet O, Rich T and Sobel A (1998) Stathmin overexpression in 293 cells affects signal transduction and the cell cycle. *FEBS Lett* **421**: 55–60

Lazar V, Diez SG, Laurent A, Giovangrandi Y, Radvanyi F, Chopin D, Bidart JM, Bellet D and Vidaud M (1995) Expression of human chorionic gonadotropin beta subunit genes in superficial and invasive bladder carcinomas. *Cancer Res* **55**: 3735–3738

Luo X-N, Mookerjee B, Ferrari A, Mistry S and Atweh GF (1994) Regulation of phosphoprotein p18 in leukemic cells. Cell cycle regulated phosphorylation by $p34^{cdc2}$ kinase. *J Biol Chem* **269**: 10312–10318

Marklund U, Osterman O, Melander H, Bergh A and Gullberg M (1994) The phenotype of a ‘cdc2 kinase target site-deficient’ mutant of oncoprotein 18 reveals a role of this protein in cell cycle control. *J Biol Chem* **269**: 30626–30635

Marklund U, Larsson N, Melander Gradin H, Brattsand G and Gullberg M (1996) Oncoprotein 18 is a phosphorylation-responsive regulator of microtubule dynamics. *EMBO J* **15**: 5290–5298

Maucuer A, Camonis JH and Sobel A (1995) Stathmin interaction with a novel putative kinase and coiled-coil forming protein domains. *Proc Natl Acad Sci USA* **92**: 3100–3104

*© 2000 Cancer Research Campaign* 

Maucuer A, Ozon S, Manceau V, Gavet O, Lawler S, Curmi P and Sobel A (1997) KIS is a protein kinase with an RNA recognition motif. *J Biol Chem* **272**: 23151–23156

Melander Gradin H, Marklund U, Larsson N, Chatila TA and Gullberg M (1997) Regulation of microtubule dynamics by $Ca^{2+}$/calmodulin-dependent kinase IV/Gr-dependent phosphorylation of oncoprotein 18. *Mol Cell Biol* **17**: 3459–3467

Melander Gradin H, Larsson N, Marklund U and Gullberg M (1998) Regulation of microtubule dynamics by extracellular signals: cAMP-dependent protein kinase switches off the activity of Oncoprotein 18 in intact cells. *J Cell Biol* **140**: 1–11

Melhem RF, Zhu XX, Hailat N, Strahler JR and Hanash SM (1991) Characterization of the gene for a proliferation-related phosphoprotein (oncoprotein 18) expressed in high amounts in acute leukemia. *J Biol Chem* **266**: 17747–17753

Nylander K, Marklund U, Brattsand G, Gullberg M and Roos G (1995) Immunohistochemical detection of oncoprotein 18 (Op18) in malignant lymphomas. *Histochemical Journal* **27**: 155–160

Pannetier C, Delassus S, Darche S, Saucier C and Kourilsky P (1993) Quantitative titration of nucleic acids by enzymatic amplification reactions run to saturation. *Nucl Acid Res* **21**: 577–583

Pasmantier R, Danoff A, Fleischer N and Schubart UK (1986) P19, a hormonally regulated phosphoprotein of peptide-hormone producing cells: secretagogue-induced phosphorylation in AtT-20 mouse pituitary tumor cells and in rat and hamster insulinoma cells. *Endocrinology* **19**: 1229–1238

Peto R, Pike MC, Armitage P, Breslow NE, Cox DR Howard SV, Mantel N, McPherson K, Peto J and Smith PG (1977) Design and analysis of randomized clinical trials requiring prolonged observation of each patient. II. Analysis and examples. *Br J Cancer* **35**: 1–39

Peyron J-F, Aussel C, Ferrua B, Häring H and Fehlmann M (1989) Phosphorylation of two cytosolic proteins. An early event of T-cell activation. *Biochem J* **258**: 505–510

Pugh BF and Tjian R (1990) Mechanism of transcriptional activation by Sp1: evidence for coactivators. *Cell* **61**: 1187–1197

Pugh BF and Tjian R (1991) Transcription from a TATA-less promoter requires a multisubunit TFIID complex. *Genes Dev* **5**: 1935–1945

Sambrook J, Fritsch EF and Maniatis T (1989) *Molecular Cloning: A Laboratory Manual*, 2nd Edn. Cold Spring Harbor Laboratory Press: Cold Spring Harbor, NY

Sarkar G and Bolander ME (1994) The ‘looped oligo’ method for generating reference molecules for quantitative PCR. *BioTechniques* **17**: 864–866

Schubart UK, Xu J, Fan W, Cheng G Goldstein H, Alpini G, Shafritz DA, Amat JA, Farook M, Norton WT, Owen TA, Lian JB and Stein GS (1992) Widespread differentiation stage-specific expression of the gene encoding phosphoprotein p19 (metablastin) in mammalian cells. *Differentiation* **51**: 21–32

Slamon DJ, Goldophin W, Jones LA, Holt JA, Wong SG, Keith DE, Levin WJ, Stuart SG, Udove J, Ullrich A and et al (1989) Studies of HER-2/neu protooncogene in human breast and ovarian cancer. *Science* **244**: 707–712

Sobel A (1991) Stathmin: a relay phosphoprotein for multiple signal transduction? *Trends Biochem Sci* **16**: 301–305

Sobel A and Tashjian AH, Jr (1983) Distinct patterns of cytoplasmic protein phosphorylation related to regulation of synthesis and release of prolactin by GH cells. *J Biol Chem* **258**: 10312–10324

Sobel A, Boutterin MC, Beretta L, Chneiweiss H, Doye V and Peyro-Saint-Paul H (1989) Intracellular substrates for extracellular signaling: characterization of a ubiquitous, neuron-enriched phosphoprotein (Stathmin). *J Biol Chem* **264**: 3765–3772

Strahler JR, Lamb BJ, Ungar DR, Fox DA and Hanash SM (1992) Cell cycle progression is associated with distinct patterns of phosphorylation of Op18. *Biochem Biophys Res Commun* **185**: 197–203

 *© 2000 Cancer Research Campaign*
